# Engagement in Physical Activity Improves after Participation in Pasos Para Prevenir Cancer—An Obesity-Related Cancer Prevention Program in El Paso, Texas

**DOI:** 10.3390/ijerph191811607

**Published:** 2022-09-15

**Authors:** Roy Valenzuela, Stefan Saadiq, Sandra Cobos, Jennifer J. Salinas

**Affiliations:** 1 Department of Molecular and Translational Medicine, Paul L. Foster School of Medicine, Texas Tech University Health Sciences Center El Paso, El Paso, TX 79905, USA; 2 Francis Graduate School of Biomedical Sciences, Texas Tech University Health Sciences Center El Paso, El Paso, TX 79905, USA

**Keywords:** obesity, cancer, underserved, physical activity

## Abstract

Background: This present study experimentally evaluated the Pasos Para Prevenir Cancer (PPPC) program to determine whether participation was associated with improved physical activity engagement. Evidence suggests that obesity prevention programs improve physical activity (PA) engagement and lead to healthier weights, which substantially impacts cancer and cardiometabolic disease risk. There is a shortage of knowledge on the effectiveness of programs tailored to populations along the U.S.–Mexico border. Methods: We collected demographic, nutrition, and physical activity data at baseline, 6 months, and 12 months using the Research Electronic Data Capture (REDCap) for 209 participants. We analyzed the average metabolic equivalents (METS) per week for all physical activity levels and types and the achievement of the recommended METS per week to determine the demographic characteristics most associated with a change between baseline, 6 months, and 12 months. Results: Light activity was the most common activity at all three points, and it slightly increased at 6 months in work settings. Subjects conducted moderate physical activity primarily at home and work, and moderate physical activity increased more compared to vigorous physical activity. Conclusions: Intervention tailoring might improve PA engagement in Mexican Americans residing on the U.S.–Mexico border; however, larger studies that are more diverse are required.

## 1. Introduction

Mexican Americans suffer disproportionately from obesity-related cancers [1]. This disparity is no truer than on the Texas–Mexico border (TMB), where, for example, liver cancer rates are more frequent in the Mexican-American population than in any other region in the U.S.A and other LatinX groups [2]). Obesity-related cancers along the TMB are of concern, since this region is medically underserved, which creates barriers to cancer care [2]. These barriers have contributed to higher rates of late-stage cancer diagnoses [1,3] and poorer cancer outcomes [4]. Therefore, primary prevention programs that help reduce the incidence of obesity-related cancers are likely to be the most cost-effective [5] and broad-reaching strategy [5] to reduce the impact on this health disparate population.

There are approximately 2.6 million Mexican Americans residing in the 32 counties along the Texas–Mexico border, representing 7% of the total Texas population [6,7]. El Paso, the location of this current study, is the largest city on the Texas–Mexico border [8]. Nearly 40% (37.8%) of its residents are obese due to high levels of sedentary time (30.7% low physical activity engagement and 25.4% inactive) [9]. Low engagement in physical activity is often precipitated by a lack of resources to be physically active; however, 81.8% of El Paso residents live close to a park or recreational facility [7,10], suggesting that access may not be the primary barrier to physical activity engagement in El Paso. The most current Texas Behavioral Risk Factor Surveillance System (BRFSS) indicated that 74.6% of El Pasoans partook in some type of physical activity monthly, compared to 70.2% of the Texas statewide population, and 57.9% of the U.S. Hispanic population [11].

There is substantial evidence that education programs improve physical activity engagement and lead to healthier weights thereby reducing the risk of cancer and cardiometabolic diseases [12,13,14,15,16,17,18,19,20,21,22,23]. In general, programs have been delivered to other Latinx populations including families, children, and women at schools, community centers, and clinics [12,13,14,15]. However, effective strategies have been primarily established for low socioeconomic Latinx populations, leaving a gap in knowledge on effective strategies in economically diverse populations, such as El Paso. While El Paso is an ethnically homogenous population, it is socioeconomically diverse [24,25]. The purpose of this study is to provide initial evidence that a tailored intervention adapted to the needs and interests of diverse socioeconomic Latinx populations will be associated with improvements in physical activity engagement. In this paper, we describe findings from our evaluation study and provide implications for future work in improving physical activity on the U.S.–Mexico border using a tailored approach.

## 2. Materials and Methods

### 2.1. Setting

El Paso, Texas, is located in the far west of Texas bordering the U.S. state of New Mexico and the Mexican state of Chihuahua. The county is large at 1015 miles² and has a population of 865,657, with 82.9% being LatinX/Hispanics, predominantly Mexican Americans [26]. The median household income is USD 46,871 (U.S. USD 62,843), and 18.8% (U.S. 10.5%) live in poverty (23). El Paso is also deemed medically underserved by HRSA, [27] which is a barrier to access to quality healthcare.

### 2.2. Recruitment and Inclusion Criteria

During educational sessions, health educators invited participants to participate in the program evaluation. PPPC staff contacted participants who had agreed to participate in the study via phone. During that call, the participants consented to the study and agreed to complete the survey by phone or online through the REDCap survey portal. In total, 209 participants were included in this study (see Table 1). Inclusion criteria for this study included participants who completed all three data points (baseline, 6-month, and 12-month surveys, were 18 years of age or older, lived in El Paso County, Texas, and were not pregnant. Exclusion criteria included participants who did not complete all three data points (missed the 6- or 12-month survey), subjects who lived outside of El Paso County, or anyone pregnant. The participants ranged from 18 to over 60 years of age, were primarily women (87%), and were born in Mexico (51%) (See Table 1). Additionally, in Table 1, the majority of the sample was married (64%), the majority had at least a high school diploma (78%), and 45% spoke Spanish at home.

### 2.3. Pasos Para Prevenir Cancer Education Program

The Pasos Para Prevenir Cancer (PPPC) curriculum was developed through an exhaustive review of existing evidence-based programs [19,20,21,22,28]. The topics covered by the PPPC are the 13 obesity-related cancers, measures of obesity, goal setting, nutrition, and physical activity. The five-session program lasted approximately one hour and included an exercise or cooking demonstration. Program participants were recruited through partnering organizations such as schools, city departments, churches, senior centers, and employers. Partner organizations selected to receive the entire 5-session curriculum or selected other options to tailor to the needs of their organization and clientele [25]. Tailoring included language (English/Spanish), number of sessions, education with cooking or physical activity demonstration, and supplemental information on food and physical activity, such as superfoods or foods to eat when exercising. Evaluation study participants were recruited after completing the first session. Program participants were contacted by phone by a research assistant who provided information about the study and obtained consent.

### 2.4. Measurement

Data collection occurred using REDCap, a secure web application for creating and governing online surveys and databases. Surveys can be used online using the Online Designer or offline using a Microsoft Excel template. For this study, health educators or the lead analyst collected self-reported data via phone calls or face-to-face interviews. The authors collected data on height, weight, physical activity engagement, and nutrition at baseline, 6 months, and 12 months. Height was recorded in inches, while weight was recorded in pounds (lbs), and both physical activity and nutrition questions were manually inputted based on participants’ responses. Each participant received an incentive for each survey conducted. Participants had a choice between a water bottle, measuring cups, resistance band, and an apron for completing the baseline survey. They would then receive a swift card ($12.00) for completing each of the six and 12-month surveys.

### 2.5. Outcome Variables

#### Physical Activity

The authors of this study used the International Physical Activity Questionnaire (IPAQ) to measure physical activity engagement [26]. The IPAQ is a widely used physical activity inventory intended to assess participants’ light, vigorous, and moderate physical activity [29]. The IPAQ is available in English and Spanish in a long or short form. Both the IPAQ short and long forms have been used to measure physical activity levels in Mexican-American populations. A study comparing physical activity levels between two U.S.–Mexico Border counties indicated that the IPAQ had a reliability of 0.80 and validity of 0.30 when used in international studies. The questions were designed to detect overall physical activity, during work, transportation, housework, and leisure time, as well as time spent sitting by asking participants what type of activities they conducted and for how long. Additionally, participants filled out the form online or over the telephone with the aid of a health educator. For this study, MET minutes per week were calculated for light, moderate, and vigorous activity. Light activity was classified as anything less than 600 MET min per week, moderate activity was classified as 600 to 3000 MET min per week, and vigorous activity levels were classified as anything >3000 MET min per week.

We used the long form, which contained five parts including job-related PA, transportation PA, housework, house maintenance, caring for family, recreation, sport, leisure-time PA, and time spent sitting. Job-related PA included paid activities such as farming, volunteer work, course work, and other unpaid work conducted outside their home. Vigorous PA included activities such as heavy lifting, digging, heavy construction, or climbing stairs, which were performed as part of work and for at least 10 min at a time. Moderate PA included activities such as carrying light loads as part of work for at least 10 min at a time. Job-related light PA included walking for at least 10 min at a time as part of work. Transportation PA was based on how an individual traveled from place to place including to work, shopping, and other areas. The survey asked how many days a person rode on a motorized vehicle and for how long. It also asked whether a person either rode a bike or walked, as a form of transportation to and from places, for how many days and for how much time.

### 2.6. Demographic Confounding Variables

Demographic characteristics were collected at baseline. The variables included age (18–39, 40–59, 60+), sex, married (yes/no), high school diploma or above (yes/no), birthplace (U.S. born, Mexico, other), and language use at home (English, Spanish, other).

### 2.7. Data Analysis

Analysis was completed to determine the normalcy of distributions and missing data. Since data collection is ongoing, a determination was made as to which participants were eligible to complete all data collection points (baseline, 6 months, and 12 months). Intent to treat was used to address missing data for eligible participants. We generated the baseline characteristics first, followed by average METS per week by activity type for the baseline, 6 months, and 12 months. Finally, we analyzed meeting METs per week guidelines for moderate physical activity by demographic characteristics. The Texas Tech University Health Sciences Center El Paso (TTUHSC EP) Institutional Review Board (IRB) (E19077) approved this evaluation study. The participants completed and signed an informed consent, which included consent to include their data in aggregated analysis and publication.

## 3. Results

**Figure 1:**
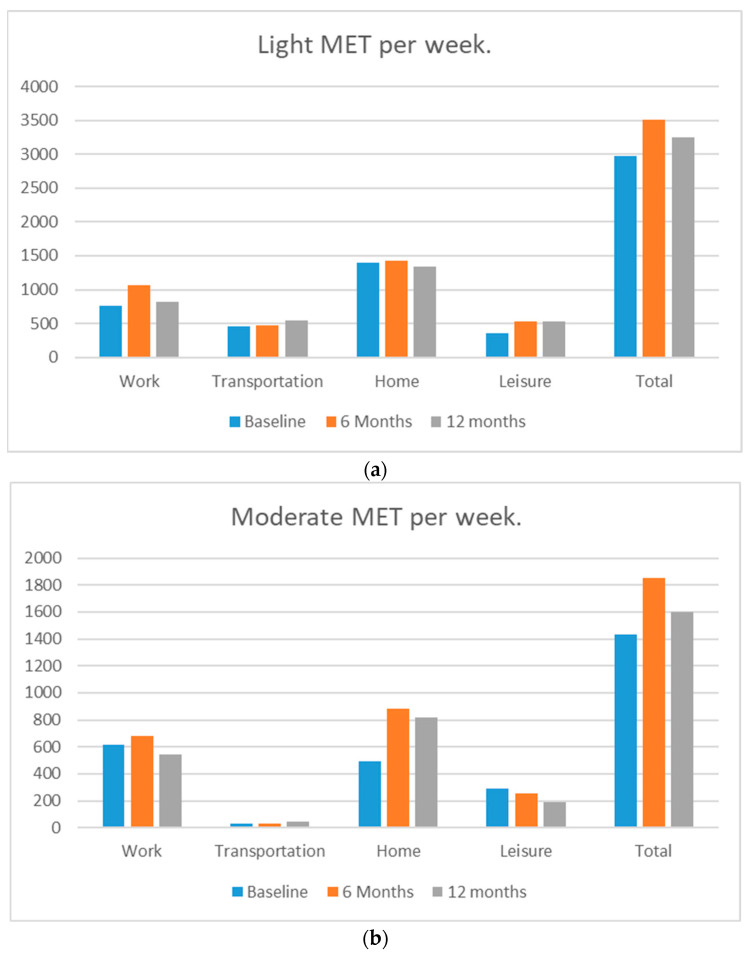

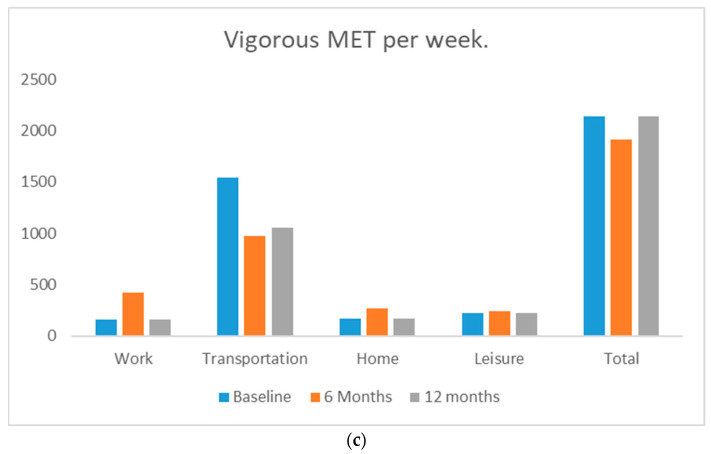
MET minutes per week of light, moderate, and vigorous physical activity. (**a**) Light MET per week; (**b**) moderate MET per week; and (**c**) vigorous MET per week.

Figure 1 presents the MET minutes per week of light, moderate, and vigorous physical activity. This figure presents the average by intensity and source of activity (work, home, leisure time, and total). Light activity (Figure 1a) was the most common activity at all time points. The greatest contribution to light activity was achieved at home. There was a small increase in light activity at 6 months, which we observed primarily in work settings. Participants most commonly achieved moderate activity (Figure 1b) at work or in their homes. Participants increased and sustained moderate activity at home primarily, which provided the greatest contribution to overall moderate activity improvements after PPPC participation. Overall, we observed greater increases in moderate physical activity compared to vigorous activity (Figure 1c). Transportation was the most common vigorous activity and changed the least throughout the follow-up period. It is important to note that these data were collected during the COVID-19 pandemic, which may explain the increases in home and not leisure, since many public and private venues for exercise were closed during this time.

Table 2 presents the met moderate physical activity (MPA) guidelines of 3000 MET minutes per week by baseline demographic characteristics. At baseline, there was little difference in meeting MPA guidelines by demographic characteristics. The one exception was age, where participants who were 60 years or older had a smaller proportion that met the MPA guidelines compared to their younger counterparts. However, at 6 months, the participants 60 years and older closed the guideline gap and maintained this improvement through to 12 months. Those with less than a high school diploma substantially improved MPA engagement and maintained this level of activity at 12 months. Finally, those born in Mexico and who spoke primarily Spanish at home had notable increases, although not significant at both follow up time points.

## 4. Discussion

El Paso, along with other areas along the U.S.–Mexico border, is unique in that most who inhabit this region are Mexican Americans speaking both Spanish and English [30]. Most evidence-based educational programs have been tested and delivered to non-border older adults [30,31,32]. This study provides new information on behavioral intervention tailoring in PPPC that resulted in modest but sustained increases in vigorous, moderate, and light physical activity up to 12 months after program participation. Improvements were most observed in moderate activity. Improvements in meeting guidelines for activity were observed primarily among older adults, non-high school graduates, immigrants, and those speaking primarily Spanish in the home. Moreover, a significant proportion of these participants improved their meeting moderate physical activity guidelines (3000 MET minutes per week) at 6 months and sustained their engagement at 12 months.

Tailoring to meet the needs of a socioeconomically diverse Mexican-American population is a promising approach to improving behaviors that reduce the risk of cancer and other chronic diseases. Due to the small participant size and the evaluation nature of this present study, a larger-scaled clinical trial should be the next step in determining approach effectiveness and under what circumstances. Additionally, there should be a focus on what further adaptations may be needed to improve the response among younger, higher educated, and U.S.-born Mexican Americans. The PPPC was tailored to reach bilingual audiences of all ages. For example, younger adults of working age may be more inclined to improve their activity at work since much of their walk-time is spent at their job sites, whereas older adults may be more likely to improve their activity at home rather than in other settings due to more time being spent at home. These tendencies may explain the observed patterns of physical activity engagement by intensity in our evaluation study.

An important pattern to note is the observed change in activity type and intensity. Vigorous activity regardless of the data collection point occurred primarily through transportation. Moderate and light activity occurred primarily at work and home. Most evidence-based interventions that are intended to improve physical activity are designed to target both moderate and vigorous activity [33]. While most provide education on how and why to improve activity, special consideration should also be made to how and where physical activity is most likely to occur by the target population. Most intervention programs target Mexican Americans and other Latino groups that are community-based and focus on children and families [33,34,35]. While these programs are important in setting up lifelong behaviors in children, there is a substantial gap in knowledge in effective approaches to improve engagement among adults without young children and older adults. For example, there is substantial literature on work-based interventions in non-border populations [36,37,38,39,40,41,42,43,44,45,46,47,48,49,50,51,52,53,54,55,56,57]. This may be an important direction to move as work appears to be a setting in which our participants from the U.S.–Mexico border engage in a significant amount of their daily physical activity.

Previous work on transportation-related physical activity has largely focused on encouraging active transportation through bike use, public transit, and walking school buses [58,59,60]. These efforts have largely occurred through policy changes, suggesting that vigorous activity promotion strategies may be most effective at this level [61,62,63,64,65,66,67,68]. Therefore, future interventions that are intended to improve physical activity should take into consideration age and policies in place, in addition to language and culture tailored to the needs of the target group. Multiple strategies to improve engagement may be needed to maximize response and to improve the population’s physical activity engagement. For example, work-based approaches may yield better responses for working-aged adults [69]. Home-oriented approaches may be better at improving older adult responses. Policies to support active transportation may be the best approach to improving vigorous physical activity engagement [70]. More research is needed to better understand how to optimize physical activity engagement using multiple strategies at different levels to achieve the desired effect in unique populations such as that on the U.S.–Mexico border, where the need is high, and the resources are low.

Pasos Para Prevenir Cancer (PPPC) utilizes evidence-based education content to improve physical activity engagement, nutrition, and obesity outcomes. This evaluation study provided initial evidence that education tailored to address specific learning needs in socioeconomically diverse Mexican American populations may be effective in improving physical activity engagement in home and work settings. Since this study was an evaluation of program implementation effectiveness, several limitations should be mentioned. First, the study participants were predominantly female (86.53%). Efforts should be made to improve the recruitment of men into the program evaluation. Additionally, this was an evaluation study to determine implementation effectiveness using a tailored approach. A larger randomized trial should be conducted to determine the true effect of this tailored intervention in leading to meaningful changes in physical activity engagement. Moreover, since PPPC was implemented in El Paso, Texas, in the U.S.–Mexico border region, it is not clear if a similar approach would work elsewhere. Finally, this program was implemented during the COVID-19 pandemic. The program had to adjust to stay-at-home orders and social distancing throughout its implementation. This may have impacted the response to the intervention because of limitations due to the Stay Home, Work Safe orders. Future evaluation should be conducted when El Paso has returned to pre-pandemic levels of activity.

## 5. Conclusions

The results from this evaluation study suggest that using a tailored intervention approach may be effective in improving physical activity engagement in populations that are ethnically homogenous but socioeconomically heterogeneous, leading to improvements in overall physical activity engagement. Future studies should examine intervention tailoring as an implementation approach for behavioral changes in border and other Latinx populations who are at disparate risk for cancer and other chronic diseases related to physical inactivity and sedentary lifestyles.

## Figures and Tables

**Table 1:** Demographic characteristics of the PPPC evaluation study participants.

Sex		
Male	28	13%
Female	181	87%
**Age**		
18–39	63	30%
40–59	90	43%
≥60	56	27%
**Married**		
No	76	36%
Yes	133	64%
**H.S Diploma**		
No	46	22%
Yes	163	78%
**Birthplace**		
USA	98	47%
Mexico	107	51%
Other	4	2%
**Language use at home**		
English	52	25%
Spanish	95	45%
Other	62	30%

**Table 2:** Participants who met the guidelines for moderate physical activity by demographics.

	Baseline			6 Months			12 Months		
**n(%)**	**Meets**	**Does Not Meet**	***p*-Value**	**Meets**	**Does Not Meet**	***p*-Value**	**Meets**	**Does Not Meet**	***p*-Value**
**Sex**									
Male	3 (10.7)	25 (89.3)	0.77	3 (10.7)	25 (89.3)	0.25	3 (10.7)	25 (89.3)	0.22
Female	23 (12.7)	158 (87.3)		36 (19.9)	145 (80.1)		37 (20.4)	144 (79.6)	
**Age**									
18-39	11 (17.5)	52 (82.5)	ref	12 (19.0)	51 (81.0)	ref	9 (14.3)	54 (85.7)	ref
40-59	13 (14.4)	77 (85.6)	0.61	14 (15.6)	76 (84.4)	0.57	20 (22.2)	70 (77.8)	0.22
60+	2 (3.6)	54 (96.4)	0.03	13 (23.2)	43 (76.8)	0.58	11 (19.6)	45 (80.4)	0.44
**High school graduate**									
Yes	21 (12.9)	142 (87.1)	0.72	24 (14.7)	139 (85.3)	0.006	26 (16.0)	137 (84.0)	0.03
No	5 (10.9)	41 (89.1)		15 (32.6)	31 (67.4)		14 (30.4)	32 (69.6)	
**Married**									
Yes	20 (15.0)	113 (85.0)	0.13	24 (18.0)	109 (82.0)	0.763	26 (19.6)	107 (80.4)	0.84
No	6 (7.9)	70 (92.1)		15 (19.7)	61 (80.3)		14 (18.4)	62(81.6)	
**Nativity**									
United States	15 (15.3)	83 (84.7)	ref	16 (16.3)	82 (83.7)	ref	13 (13.3)	85 (86.7)	ref
Mexico	10 (9.4)	97 (90.6)	0.2	22 (20.6)	85 (79.4)	0.44	26 (24.3)	81 (75.7)	0.05
Other	1 (25.0)	3 (75.0)	0.61	1 (25.0)	3 (75.0)	0.65	1 (25.0)	3 (75.0)	0.51
**Language use**									
English	7 (13.5)	45 (86.5)	ref	6 (11.5)	46 (88.5)	ref	8 (15.4)	44 (84.6)	ref
Spanish	12 (12.6)	83 (87.4)	0.89	23 (24.2)	72 (75.8)	0.07	22 (23.2)	73 (76.8)	0.27
Other	7 (11.3)	55 (88.7)	0.73	10 (16.1)	52 (83.9)	0.48	10 (16.1)	52 (83.9)	0.91

## Data Availability

Not applicable.
